# Prevalence and gender patterns of mental health problems in German youth with experience of violence: the KiGGS study

**DOI:** 10.1186/1471-2458-13-628

**Published:** 2013-07-02

**Authors:** Robert Schlack, Franz Petermann

**Affiliations:** 1 Department of Epidemiology and Health Monitoring, Robert Koch Institute, General-Pape-Strasse 62-66, 12101 Berlin, Germany; 2 University of Bremen, Center of Clinical Psychology and Rehabilitation, Grazer Strasse 2-6, 28334 Bremen, Germany

**Keywords:** Youth violence, Mental health, Emotional problems, Conduct problems, ADHD, Disordered eating behaviors, Somatic problems, Substance use, Gender, Representative study

## Abstract

**Background:**

Research examining mental health in violence-affected youth in representative samples is rare. Using data from the nationally representative German Health Interview and Examination Survey for Children and Adolescents (KiGGS) this study reports on gender-specific prevalence rates and associations of a broad range of internalizing and externalizing mental health problems: emotional problems, conduct problems, ADHD, disordered eating, somatic pain and substance use in youth variously affected by violence. While internalizing is generally more common in girls and externalizing in boys, observations of prior non-normative studies suggest reverse associations once an individual is affected by violence. The occurrence of such “gender cross-over effects” is therefore examined in a representative sample.

**Methods:**

The sample consisted of 6,813 adolescents aged 11 to 17 from the German Health Interview and Examination Survey for Children and Adolescents (KiGGS): Applying multivariate logistic regression analyses, associations between each type of violence history and mental health indicator were determined for perpetrators, victims, and perpetrating victims of youth violence. Moderating effects of gender were examined by using product term interaction.

**Results:**

Victim status was associated primarily with internalizing problems, while perpetrators were more prone to externalizing problems. Perpetrating victims stood out with respect to the number and strength of risk associations with all investigated mental health indicators. However, the risk profiles of all violence-affected youth included both internalizing and externalizing mental health problems. Gender cross-over effects were found for girls and boys: despite lower overall prevalence, girls affected by violence were at far higher risk for conduct problems and illicit drug use; by contrast, somatic pain, although generally lower in males, was positively associated with perpetrator status and perpetrating victim status in boys. All violence-affected youth exhibited significantly higher rates of cumulative mental health problems.

**Conclusions:**

The results highlight the importance of violence for the mental health of youth. They reveal a particular vulnerability as a function of gender. Implications for policy making, clinical practice and research are discussed.

## Background

Studies of mental health problems in the general child and adolescent population of western industrialized countries indicate that 9.5% to 22.2% of youth suffer from one or more mental disorders [1,2]. Although representative, population-based studies are largely absent in the literature, there is evidence that the prevalence of mental health problems in youth who are involved in such deviant behaviors as youth violence might be much higher than in the general population [3]. Youth violence can take several forms: peer aggression – including school bullying and dating violence – but also more severe acts such as robbery, assault or date rape, each of which can result in serious emotional harm, physical injury or even suicide and death [4-6]. A current definition of youth violence posits that a young person “can be a victim, an offender, or a witness to the violence” [4] p.1. However, in bullying research, for example, it is widely acknowledged that an individual may be a victim and a perpetrator at the same time. Those who both perpetrate and are victimized have been variously termed throughout the literature (e.g. aggressive victims, bully-victims or perpetrating victims) and are regularly found with the lowest levels of psychosocial functioning [7,8]. Recent research suggests that it might be reasonable to adopt this category in research on youth violence [9,10].

Child and adolescent mental health problems are traditionally conceptualized as internalizing and externalizing emotional and behavioral problems. Externalizing problems are characterized by dysregulated behaviors, which include problems with inhibiting unwanted behavior, controlling attention and cognitive processing. They include attention deficit hyperactivity disorder (ADHD), oppositional defiant disorder (ODD), and conduct disorder (CD). By contrast, internalizing problems are associated with an inability to control negative emotionality, such as rumination, loneliness, sadness, anxiety, and depression [11]. Externalizing problems are consistently found to be more common in boys, whereas internalizing problems are more prevalent in girls [2,12]. Violent behaviors in adolescence are related to both internalizing and externalizing mental problems [13].

### Internalizing problems and violent behaviors

Violent victimization has been linked with higher risks of internalizing, such as anxiety and depression, low self-control, and specific forms of anxiety disorders [14-16]. Similarly, disordered eating behaviors in adolescence might represent internalizing rather than externalizing problems [17]. Research examining eating disorders in the context of youth’s violent behaviors is scarce. Ackard and Neumark-Sztainer [5] found evidence of a relation between disordered eating behaviors and dating violence in youth. Two other studies found increased likelihoods of eating disorders in young female adults who retrospectively reported peer victimization at school [18,19]. Furthermore, somatic syndromes may be regarded as internalizing problems. Somatic syndromes include pain, fatigue, sleep disturbance, and cognitive impairment and are associated with exposure to stress [20]. Although violence involvement clearly constitutes a stressful event, only few studies have examined the associations between violence and somatic complaints in youth. For example, Piko et al. [21] found that physical aggression was associated with increased psychosomatic symptoms. Also, sleeping problems have been linked with anger, impulsivity and aggression in male juvenile offenders; sleep problems have also been connected with victimization experiences [22,23].

### Externalizing problems and violent behaviors

Externalizing problems, such as CD, ODD, delinquency and antisocial personality traits, are associated with violence perpetration [24,25]. Similarly, young people with ADHD have an elevated risk of being perpetrators of violence, as ADHD is often comorbid with CD and ODD [26]. However, controlling for CD, Fang and colleagues [25] showed that ADHD uniquely predicted violence perpetration. However, as young people with ADHD exhibit maladjusted behaviors – such as fidgeting, running around when expected to sit still, or interrupting or intruding on others (for full diagnostic criteria, see [27]) – they are also likely to be the targets of peer aggression. In a Swedish school study, Holmberg and Hjern [28] found that youth with ADHD had a more than tenfold increased risk of being victimized.

Although related to both violence perpetration and violent victimization [9,29], substance use may be considered an externalizing rather than an internalizing behavior, specifically in the context of violence. For example, violent adolescents tend to drink alcohol to reduce the negative consequences of their multiple problems or to conform to group norms that promote alcohol use or violent behavior [30]. In a high-risk sample of youth at risk for school drop-out, marijuana, hard-drug and poly-drug use was more strongly associated with perpetration than with victimization experiences [9]. Nonetheless, longitudinally, physical victimization in juveniles was associated with a 60% increase in the relative risk of alcohol consumption and a more-than-doubled risk of marijuana and inhalant use [29].

### Gender differences

For both social and biological reasons, internalizing disorders are more frequent in girls, while externalizing disorders are more prevalent in boys. Hankin and Abramson [31] suggested a cognitive vulnerability-transactional stress depression model to specifically explain why adolescent girls are more likely to develop internalizing symptoms than adolescent boys. According to the model, the occurrence of initial interpersonal negative life events accounts for increases in negative affect and/or depression. As girls are more strongly focused on self-regulation and sensitivity to interpersonal relationships by dint of their socialization and thus display greater affiliative needs, they are at a higher risk for interpersonal negative events than boys [32]. Also, girls are more prone to cognitive vulnerabilities such as rumination and negative inferential style [33,34]. A number of further biological, personality and environmental reasons – such as earlier sexual maturation [35], hormonal changes associated with menarche [36], greater proneness to neuroticism [37], and a greater likelihood of being sexually abused [38] – puts girls at higher risks for both cognitive vulnerabilities and the occurrence of initial negative events. On the other hand, there are dispositional and socializational reasons why boys are more prone to externalizing problems than girls. Differences in aggression, for example, may arise from differential fetal testosterone exposure see [39], as well as from socialization practices that emphasize independence, self-assertion and autonomy, and underemphasize empathy and self-regulation in males [40,41]. Accordingly, throughout the literature, boys are found to be more likely to be involved in severe acts of violence than girls [24,40].

Nonetheless, research indicates that mental health problems – including externalizing problems – co-occur more frequently in committed girls than boys. In particular, this co-occurrence has been demonstrated with respect to disruptive behavior disorders, angry-irritable moods, somatic complaints, anxiety disorders, depression, and suicidal ideation [2,42,43]. However, the presence of real comorbidity has been denied because the majority of studies has been conducted with non-normative samples that include clinically referred or detained youth [24]. Remarkably, some studies show reverse associations of gender with both internalizing and externalizing mental health problems, once an individual is affected by violence. For example, two recent community-based studies showed that – despite a lower overall prevalence of externalizing problems in girls – those girls who were exposed to violence reported significantly higher levels of externalizing problems than boys [44,45]. Conversely, boys who were the victims of date violence and/or date rape have been found to be at considerably higher risks for disordered eating, such as binge eating, vomiting and laxative use [5], although eating disorders are generally more common in girls [46]. Studies that have observed such “gender cross-over effects” have not usually explicitly addressed or discussed this phenomenon. A potentially related observation, termed “gender paradox”, has been described earlier in a review of research on conduct disorder and associated comorbidities [47]. The gender paradox refers to the fact that “despite the lower prevalence of disruptive disorders, girls who suffer from CD are at higher risk for developing […] comorbid conditions“ [47] p. 517. However, the gender paradox has been described as a condition of girls only. Yet, there is evidence that gender cross-over effects might not be limited to just one sex, as demonstrated above.

### Study aims

As stated above, most of the previous research on violence and mental health in youth has been conducted with non-normative samples. Also, many studies are limited to the consideration of only one or two mental health indicators at a time. Because it includes both an extensive mental health assessment and a violence assessment, the nationally representative German Health Interview and Examination Survey for Children and Adolescents (KiGGS) offers an excellent opportunity to investigate a broad range of internalizing and externalizing mental health problems in violently behaving youth. To our knowledge, no such data have been reported for Germany before. The data set makes it possible to include each of the four indicators of internalizing and externalizing disorders – emotional problems, disordered eating behavior, somatic pain, and insomnia problems – as well as conduct problems, ADHD, frequent alcohol consumption, and the habitual use of illegal drugs. The study contributes to the literature on youth violence by using an extended typology of adolescent violence involvement borrowed from research on bullying: youth with a history of perpetration only, youth with a history of victimization only, youth with a history of both perpetration and victimization, and youth with no history of violence. Our study has the following aims: *First*, to report on prevalence rates and distributions of internalizing and externalizing mental health problems by gender and the various types of adolescent violence involvement. *Second*, to multivariately examine associations of gender and mental health in violence-affected youth. An important contribution of our study is that we explicitly address the issue of potentially reverse gender stereotypes in the face of violence-affection. Specifically, a corroboration of such effects in a representative study would be highly informative for prevention, intervention and treatment efforts in order to frame more gender-specific programs. *Finally*, we were interested in how frequently youth variously involved in violence report cumulative mental health problems.

## Methods

### The KiGGS study

The German Health Interview and Examination Survey for Children and Adolescents (KiGGS) is part of the German health-monitoring system established at the Robert Koch Institute, Berlin, on behalf of the German Federal Ministry of Health [48]. The KiGGS study is conceptualized as repeated survey including both cross-sectional and longitudinal components [49]. The first replication of the survey is currently in progress. A total of 17,641 children and adolescents between the ages of 0 and 17 and their parents participated in the baseline assessment that took place between May 2003 and May 2006. The net response rate was 66.6%. The study is unique in Europe in terms of its sample size, its age range and its response rate, as shown by the EU Health Surveys Information Database (http://www.euhsid.org/). The participating children and adolescents were given a physical examination; the parents – from age 11 on, also the children and adolescents themselves – completed extensive self-administered questionnaires on their physical, social and mental health. The present study was conducted on 6,813 adolescents (3,492 boys and 3,321 girls; unweighted totals) aged 11 to 17 who took part in a violence assessment. The sampling strategy of the KiGGS study has been described elsewhere in detail [50,51]. Briefly, the sampling frame followed the principles of a stratified multistage probability sample [52]. The participants were recruited in two steps. In the first step, 167 study locations (primary sample units or PSUs) were systematically chosen from an inventory of German communities stratified according to the BIK classification [53], which measures the degree of urbanization and geographic distribution. Using the Cox procedures for community sampling [54], the number of PSUs per stratum was determined with a sampling probability proportional to population size. In the second step, an equal number of study subjects per birth cohort over the entire age range was randomly selected (simple random sample) from the local population registries. Parents of eligible children and adolescents were contacted by letter and invited to participate. Information was provided about the type of investigation, ethical approvement, data processing, the voluntary nature of participation, and monetary compensation. The study fully complies with the Declaration of Helsinki and was ethically approved by the *Charité Unversitätsmedizin, Berlin* ethics committee (no.: 101/2000) and the Federal Office for the Protection of the Data (no.: IV-401/008#0008). Written informed consent was obtained from the primary caregivers of all participants and, in addition, from participants who were 14 years and older. For more details on the objectives, design, procedures and measurements of the KiGGS study, please refer to Kurth et al. [51].

### Measures

The KiGGS study provides a basis for a variety of mental health measures. Due to constraints on time and resources, written questionnaires were used rather than diagnostic interviews. However, they included internationally recognized and validated screening tools for child mental health, an assessment of psychiatric diagnoses by clinicians (as reported by the parents), and an assessment of symptoms and behaviors self-reported by the participating youth. We intended to approximate clinically relevant symptomatology in this study. Thus, where available, clinical cut-offs of our measures were used. Below, we describe our measures by thoroughly evaluating their strengths and limitations.

### Violence involvement

Violence involvement was assessed based on two questions pertaining to the respondents’ experiences as victims of violence (“How often have you been a victim of violence in the past 12 months?”) or as a perpetrator (“How often have you been a perpetrator of violence in the past 12 months?”) [55]. The response options were *never/once/several times*. In this study, the following violence typology was chosen: youth who reported having been a victim once or more often and not having been a perpetrator were classified as victims; youth who reported having been a perpetrator of violence once or more often but who reported not having been victimized were classified as perpetrators; those who reported both victimization and perpetration once or more often were classified as perpetrating victims. Youth who did not report any violence involvement were classified as uninvolved.

### Emotional and conduct problems

Emotional and conduct problems were measured using the two relevant subscales of the parent-rated Strengths and Difficulties Questionnaire (SDQ) [56]. Emotional problems (α = .69) include items concerning anxiety and depressiveness (“Often unhappy, depressed or tearful“, “Many fears, easily scared “). The items on the conduct problems subscale (α = .58) refer to oppositional, aggressive, and antisocial behavior. Item examples include: “Generally well behaved, usually does what adults request” (to be reversed), “Often fights with other youth or bullies them”, “Steals from home, school or elsewhere”. Each subscale contains five items; all items are to be answered on a 3-point Likert scale ranging from *not true* to *certainly true*. To approximate a clinically relevant symptomatology, items of each scale were summed and dichotomized according to the German cut-off values (both emotional and conduct problems ≥ 5) [57] for the purpose of contrasting clinical vs. non-clinical ranges.

### Disordered eating behaviors

Disordered eating behaviors were assessed using the internationally recognized and validated SCOFF questionnaire [58]. It comprises five items (response options: *yes/no*) on the presence of core symptoms of anorexia and bulimia nervosa (such as vomiting, fear of losing control over eating, intensive dieting, and body weight concerns) over a period of three months. The recommended cut-off of two out of five questions answered in the affirmative was used to determine the presence of disordered eating behaviors. It must be emphasized that the questionnaire is neither designed to make diagnoses according to the Diagnostic and Statistical Manual of Mental Disorders (DSM-IV) or the International Classification of Diseases (ICD-10), nor does it discriminate between different types of disordered eating (e.g. bulimia or anorexia nervosa); rather, the survey includes disordered eating on a subclinical level. This characteristic of the questionnaire may explain the far higher prevalence rates obtained in assessments with the SCOFF questionnaire compared to the rates reported by studies that strictly apply diagnostic criteria according to the ICD-10 or DSM-IV [46].

### Somatic pain

For screening purposes, a diagnosis of somatoform pain disorder according to ICD 10 (F45.4) – “predominant complaint is of persistent, severe, and distressing pain which cannot be explained fully by a physiological process or a physical disorder”– was approximated by using information from subjects aged 11 and older regarding their predominant pain during the past three months and the frequency with which this pain occurred. The response options for this question were *once/once per month/2-3 times per month/once per week/several times per week/daily*. Predominant pain was assessed by an open category. The indicated pain localizations were initially categorized by the authors according to the ICD criteria and then counterchecked by a clinical expert in neurology and psychiatry. A symptom that occurred several times a week or daily during a 3-month period was considered persistent and frequent. If a respondent indicated that a symptom had not occurred as a consequence of a physical disease, a physiological process (e.g. growing pains or menstrual cramps), medical or dental treatment, surgery, an injury or a sports event, then the respondent was considered screen positive. Although we were able to meet most of the ICD criteria, we were unable to exclude tension headaches, and the period covered in our questionnaire was 3 rather than 6 months (as required by the ICD). The interpretation of the results should consider these restrictions.

### Sleeping problems

A screening diagnosis of insomnia was developed in collaboration with sleep medicine experts. Subjects were considered screen positive if they reported problems falling asleep (item: “I have problems falling asleep”; response options: *yes/no*) or remaining asleep (item: “I have problems remaining asleep”; response options: *yes/no*), in combination with daytime sleepiness (item: “During the last week, I was tired and weary”; response options: *often* or *always* on a 5-point Likert scale ranging from *never* to *always*). Although this constitutes a rather crude measure, it has proven useful in preliminary analyses that have distinguished youth with sleeping problems across various psychosocial dimensions [59].

### Parent-reported ADHD diagnosis

Parents were asked to indicate whether their children had ever been diagnosed with ADHD by a physician or psychologist. A subject was considered an ADHD case if his or her parents confirmed a lifetime diagnosis from a physician or psychologist [60]. In Germany, the diagnosis of ADHD is not legally restricted to child and adolescent psychiatrists or clinical child psychologists, as in other countries. Because pertinent clinical guidelines recommend referral to specialized centers or clinical psychologists in the case of suspected ADHD [61], however, it is likely that clinical diagnoses are usually assigned or at least confirmed by these professional groups. Our measure thus reflects the clinical judgments of healthcare professionals as reported by parents.

### Frequent drinking

The youth were asked to indicate whether they currently drank alcohol [62]. If they responded in the affirmative, they were then asked to indicate how much alcohol they consumed. Three common types of alcoholic beverages were presented: beer, wine (including fruit wine or champagne), and hard liquor. The youth were then asked to indicate the number of typical glasses of each beverage that they drank during a defined time period. Typical glasses were assumed to be dose equivalent. The response options ranged from *never* to *one glass or more per day* for each beverage. An index of total alcohol consumption was then calculated (range 0–21 and more glasses per week). To approximate drinking behavior with potential psychopathological relevance, we divided the index into two groups to compare an extreme group that indicated the consumption of 5 glasses or more of alcoholic beverages per week with a group with lower consumption levels. Unlike for adults, there are no recommendations as to hazardous regular alcohol consumption in adolescents in order not to promote risk-free drinking. The cut-off of 5 glasses and more was therefore arbitrarily chosen. It represents, however, alcohol consumption levels approximately at the 90^th^ percentile in our sample. The choice of cut-off is supported by a current WHO study on alcohol use and injuries in young adults that applied a categorization of weekly drinking frequencies of zero, one, 2–3, 4–5 and 6 or more glasses of alcoholic beverages [63]. In that study, a weekly consumption of 4–5 glasses already accounted for a significant increase in the likelihood of non-fatal injuries.

### Illicit drug use

Illicit drug use was assessed with 5 items addressing the use of marijuana, ecstasy, amphetamine (speed), medicinal drugs, and inhalants (e.g. glue sniffing). Youth between the ages of 11 and 17 were asked whether they had used each specific drug within the past 12 months (response options: *never/once/several times/often*), or whether they were familiar with the drug at all. An index of illicit drug use was created to classify youth as habitual users if they reported repeatedly using at least one drug (*several times/often*) during the past 12 months.

### Statistical analysis

All the statistical analyses are based on weighted data in order to represent the structure of the German child and adolescent population. In a first step, the sample weight takes account of the study design by considering both total numbers of eligible youth aged 0–17 within the PSUs and the sampling probability of the PSUs itself. The design weights are inversely proportional to the sampling probability of the study subjects, which itself is composed of the PSU’s selection probability (proportional to the number of 0- to 17-year-olds in the community) multiplied by the sampling probability of subjects within the community (i.e. the number of actual participants relative to sex and age group divided by the total number of children in the community within the respective gender and age group; the age-group classification is as follows: 0–2, 3–6, 7–10, 11–13 and 14–17). The design weighting was conducted separately for the three regions of eastern Germany, western Germany and Berlin. In a second step, the weight was adjusted for deviations from the population structure (as per December 31, 2004) regarding the cross-classification of age (in years), sex, region (eastern Germany / western Germany / Berlin), and nationality (German / not German). For further details of the weighting procedure see Kamtsiuris et al. [50]. Unless otherwise indicated, the number of cases and percentages reported in this study refers to weighted data.

The data analyses were performed using SAS (v9.3). SAS survey procedures (PROC SURVEYFREQ, PROC SURVEYMEANS and PROC SURVEYLOGISTIC) were used to account for the correlations between individuals within the clusters of the sample (PSUs). We replaced missing values using multiple imputations by Marchov Chain Monte Carlo methods as provided by PROC MI. Because the number of missing values was less than 10% on all variables, 5 sets of imputation were sufficient to reach a relative efficiency of more than .99 in all the analyses conducted. As recommended by Allison [64], we used all analysis variables and useful auxiliary variables in our data set for multiple imputation. Prior to imputation, the sample was divided by gender, imputed separately for boys and girls, and then recombined for the analyses. Combined parameter estimates and multivariate inferences were obtained using PROC MIANALYZE. Inferences for the frequency tables were obtained by unadjusted logistic regressions. For multi-category predictors, inferences are reported as ranges (min/max) of p-values. Numbers and percentages can deviate from previous descriptions of the sample due to the use of multiple imputation. Adjusted odds ratios for each violence-affected group were calculated by binary logistic regression using each mental health indicator as a dependent variable. Adjustments were made for age, socioeconomic status (*low/middle/high)*, and family structure (living with a *single parent/step-parent/birth parents/other*). The presence of moderating effects of gender for each type of violence history was examined by product term interaction. Where interaction terms were significant, we probed them using post-hoc analyses for logistic regression [65]; otherwise, they were eliminated. Finally, we examined cumulative mental health problems by calculating how often the subjects were found to have two or more co-occurring mental health problems.

## Results

### Sample description and prevalence of violence involvement

48.7% of the study subjects were girls, 51.3% were boys. The mean age was 14.12 (SE = 0.01). 26.5% came from families with a low socioeconomic status (SES), 47.1% from families with a middle SES, and 26.4% from families with a high SES. As to family structure, 13.3% lived with a single parent, 10.5% with a step parent, and 75.0% with their birth parents (other: 1.2%). According to their self-reports, 3.5% of the girls were perpetrating victims (boys: 7.4%), 9.8% (19.4%) perpetrators, and 3.5% (5%) victims of violence (all p < .001).

### Prevalence of mental health problems

The prevalence of mental health indicators in this sample has been reported on in more detail elsewhere [59,60,62,66,67]. In brief, internalizing problems – i.e. emotional problems, disordered eating behaviors, somatic pain, and sleeping problems – were consistently significantly more common among girls, whereas externalizing problems – conduct problems, diagnosed ADHD, frequent drinking, and illicit drug use – were consistently significantly more common among boys (Table 1).

**Table 1:** **Prevalence of mental health problems according to gender in German youth aged 11 to 17 (N = 7697**^a^)

	**Girls (%)**	**Boys (%)**	***p***
**Internalizing**			
***Emotional problems****(clinical range)*	11.1	8.3	*.0004*
***Disordered eating behaviors***	28.9	15.4	*<.0001*
***Somatic pain***	8.3	3.5	*<.0001*
***Sleeping problems***	7.3	4.2	*<.0001*
**Externalizing**			
***Conduct problems****(clinical range)*	5.1	7.5	*.0002*
***ADHD***	2.1	10.3	*<.0001*
***Frequent drinking***	3.1	11.9	*<.0001*
***Illicit drug use***	2.9	4.2	*.0157*
(n)	3747	3951	

Note: Inferences are based on t-values for combined sets of imputed data.

^a^ Weighted frequencies; numbers for boys and girls do not add to total due to rounding error.

Generally, the prevalence rates of mental health problems were considerably higher among subjects who indicated violence involvement. In male perpetrating victims, emotional problems, conduct problems, disordered eating behaviors, somatic pain, and frequent drinking were 1.5 to 3 times more common than among uninvolved boys. Diagnoses of ADHD were equally common in male perpetrators and male victims, whereas sleeping problems were most common in male victims. In girls, emotional problems, ADHD diagnoses, disordered eating behaviors, sleeping problems, frequent drinking, and illicit drug use were the most common in perpetrating victims; conduct problems were equally common in perpetrating victims and perpetrators. The prevalence of mental health problems was 1.5 (somatic pain) to 5 times (conduct problems and illicit drug use) higher in female perpetrating victims than in uninvolved girls (Table 2).

**Table 2:** **Prevalence of mental health problems according to history of violence and gender in German adolescents aged 11 to 17 (N = 7697**^**b**^**)**

	**Uninvolved youth (%)**	**Victims (%)**	**Perpetrators (%)**	**Perpetrating victims (%)**	***p-range***^***a***^
**Internalizing**					
***Emotional problems****(clinical range)*					
*Girls*	9.8	18.2	14.4	25.1	*<.0001*^*†*^
*Boys*	6.5	9.2	11.4	15.9	*<.0001*^*†*^
***Disordered eating behaviors***					
*Girls*	26.5	37.6	38.7	48.1	*<.0001*^*†*^
*Boys*	12.4	19.7	20.1	26.7	*<.0001*^*†*^
***Somatic pain***					
*Girls*	8.1	10.9	7.8	11.1	*.3458-.6551*
*Boys*	2.6	2.9	5.5	7.0	*.0004-.0006*
***Sleeping problems***					
*Girls*	6.2	15.9	8.8	20.7	*<.0001*^*†*^
*Boys*	3.1	9.1	5.7	6.7	*<.0001-.0004*
**Externalizing**					
***Conduct problems****(clinical range)*					
*Girls*	3.1	11.8	15.1	15.8	*<.0001*^*†*^
*Boys*	4.9	9.0	12.5	17.0	*<.0001*^*†*^
***ADHD***					
*Girls*	1.8	2.2	3.9	5.5	*.0249-.0340*
*Boys*	8.6	14.7	14.4	12.9	*.0003-.0024*
***Frequent drinking***					
*Girls*	2.7	2.4	4.5	11.9	*.0012-.0015*
*Boys*	9.5	14.5	17.5	18.2	*.0001*^*†*^
***Illicit drug use***					
*Girls*	2.0	2.0	6.2	15.3	*<.0001*^*†*^
*Boys*	2.8	4.6	7.5	7.5	*<.0001*^*†*^
*(n) Girls*	(3104)	(143)	(368)	(133)	
*(n) Boys*	(2687)	(198)	(775)	(291)	

Note: Inferences are based on Wald chi-squared tests for overall significance.

^a^ p-range: the range of p-values across the 5 imputed data sets; ^*†*^all p-values < .0001.

^b^ Weighted frequencies; numbers for boys and girls do not add to total due to rounding error.

### Multivariate associations

Violence involvement was significantly associated with all of the investigated mental health indicators in multivariate assessment. Victim status was associated with all of the internalizing variables (except somatic pain). Associations with sleeping problems were the highest for victims. Victimization was not associated with ADHD, frequent drinking, or illicit drug use; however, there were strong associations between victim status and conduct problems.

Both perpetrator status and perpetrating victim status were strongly associated with externalizing problems, i.e. conduct problems and ADHD, frequent drinking and illicit drug use. However, both perpetrators and perpetrating victims were also likely to experience emotional problems, disordered eating behaviors, sleeping problems, and somatic pain (only boys), all of which relate to internalizing problems. Nevertheless, violent youth differed in terms of the strength of these associations. Perpetrating victims showed the greatest risk associations in six of the eight investigated mental health domains—emotional problems, conduct problems, disordered eating behaviors, somatic pain (only boys), frequent drinking, and illicit drug use—compared to adolescents with a history of only perpetration or only victimization. Furthermore, they had a similarly high risk of experiencing sleeping problems as victims (Table 3).

**Table 3:** Adjusted odds ratios for the associations of different histories of violence (uninvolved, victims, perpetrators, and perpetrating victims) with the mental health problems investigated (N = 7697)

	**Uninvolved youth**	**Victims**		**Perpetrators**		**Perpetrating victims**	
		**AOR (95% CI)**	***p***	**AOR (95% CI)**	***p***	**AOR (95% CI)**	***p***
**Internalizing**							
***Emotional problems***	Ref.	1.63 (1.09-2.44)	*.0169*	1.60 (1.23-2.10)	*.0006*	2.57 (1.83-3.60)	*<.0001*
***Disordered eating behaviors***	Ref.	1.47 (1.09-1.98)	*.0121*	1.33 (1.08-1.62)	*.0063*	1.90 (1.45-2.49)	*<.0001*
***Somatic pain***	Ref.	1.37 (0.68-2.75)	*.3792*	0.99 (0.63-1.55)	*.9490*	1.40 (0.72-2.75)	*.3226*
Gender*history of violence^*†*^	Ref.	0.79 (0.22-2.81)	*.7111*	2.22 (1.17-4.22)	*.0151*	2.01 (0.86-4.72)	*.1092*
***Sleeping problems***	Ref.	2.91 (1.88-4.50)	*<.0001*	1.72 (1.27-2.32)	*.0004*	2.89 (1.91-4.37)	*<.0001*
**Externalizing**							
***Conduct problems***	Ref.	3.80 (1.93-7.46)	*.0001*	4.91 (3.12-7.73)	*<.0001*	5.06 (2.43-10.51)	*<.0001*
Gender*history of violence^*†*^	Ref.	0.47 (0.18-1.23)	*.1245*	0.53 (0.31-0.90)	*.0194*	0.68 (0.30-1.55)	*.3578*
***ADHD***	Ref.	1.56 (0.94-2.59)	*.0873*	1.82 (1.37-2.41)	*<.0001*	1.66 (1.06-2.60)	*.0268*
***Frequent drinking***	Ref.	1.49 (0.94-2.37)	*.0868*	2.21 (1.61-3.04)	*<.0001*	2.92 (1.74-4.89)	*<.0001*
***Illicit drug use***	Ref.	1.02 (0.22-4.77)	*.9802*	4.36 (2.36-8.07)	*<.0001*	8.92 (4.32- 18.43)	*<.0001*
Gender*history of violence^*†*^	Ref.	1.45 (0.24-8.77)	*.6838*	0.66 (0.32-1.34)	*.2518*	0.33 (0.12-0.87)	*.0255*

Note: *AOR* Adjusted odds ratio. Adjustment made for age, socioeconomic status (*low/middle/high*), and family structure (living with *single parent/step-parent/birth parents/other*).

^*†*^Only significant interaction terms are presented. Gender: Boys vs. girls (Ref.). History of violence: Perpetrating victims, perpetrators and victims vs. uninvolved youth (Ref.)

### Moderation results

Moderating effects of gender were found with respect to somatic pain, conduct problems and illicit drug use. Girls with any type of violence history had significantly higher risks for conduct problems and dramatically (i.e. up to 9-fold) higher risks for illicit drug use. By contrast, only male perpetrators and perpetrating victims exhibited a higher likelihood of somatic pain (Table 4).

**Table 4:** Results from the contrast analyses of significant interactions of gender and history of violence (N = 7697)

	**Uninvolved Youth**	**Victims**		**Perpetrators**		**Perpetrating victims**	
		**AOR (95% CI)**	***p***	**AOR (95% CI)**	***p***	**AOR (95% CI)**	***p***
***Somatic pain***							
Girls	Ref.	1.37 (0.68-2.75)	*.3792*	0.99 (0.63-1.55)	*.9490*	1.40 (0.72-2.75)	*.3226*
Boys	Ref.	1.08 (0.39-2.99)	*.8891*	2.19 (1.39-3.43)	*.0007*	2.82(1.57-5.07)	*.0005*
***Conduct problems***							
Girls	Ref.	3.80 (1.93-7.46)	*.0001*	4.91 (3.12-7.73)	*<.0001*	5.06 (2.43-10.51)	*<.0001*
Boys	Ref.	2.25 (1.23-4.10)	*.0081*	3.30 (2.37-4.61)	*<.0001*	4.33 (3.03-6.19)	*<.0001*
***Illicit drug use***							
Girls	Ref.	1.02 (0.22-4.77)	*.9802*	4.36 (2.36-8.07)	*<.0001*	8.92 (4.32- 18.43)	*<.0001*
Boys	Ref.	1.48 (0.59-3.75)	*.4065*	2.88 (1.87-4.43)	*<.0001*	2.94 (1.60-5.43)	*.0005*

Note: *AOR* Adjusted odds ratio. Adjustment made for age, socioeconomic status (*low/middle/high*), and family structure (living with *single parent/step-parent/birth parents/other*).

### Cumulative mental health problems

**Figure 1:**
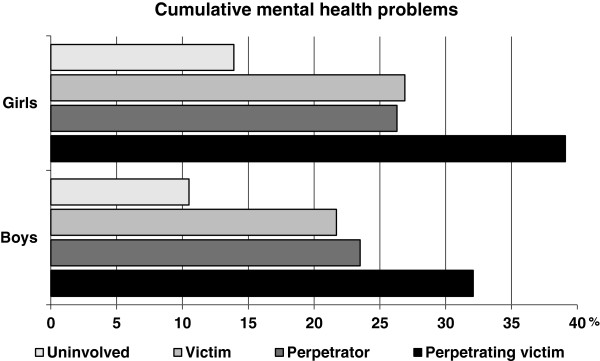
Percentage of two or more mental health problems in German youth according to violence and gender (N = 7697).

Of all participants, 15.2% of the girls and 16.5% of the boys (p-range: .1559-.2665) were found to have at least two mental conditions (data not in figure or table). Figure 1 shows that 39.1% of the female perpetrating victims (boys: 32.1%), 26.3% of female perpetrators (23.5%), and 26.9% of female victims of violence (21.7%) exhibited two or more mental health problems compared with 13.9% (10.5%) of uninvolved youth (all p < .0001 for both boys and girls).

## Discussion

The aim of this study was to report on prevalence rates and distributions of internalizing and externalizing mental health problems in adolescents by gender and various types of adolescent violence involvement – victims, perpetrators, perpetrating victims and uninvolved youth – in a nationally representative sample. We further aimed to assess multivariate associations of gender and mental health in violence-affected youth aged between 11 and 17. Specifically, we were interested in determining whether reverse associations of gender and mental health would occur in a representative sample, something that had occasionally been observed in prior non-normative studies [5,44,45]. Finally, we aimed to explore the presence of cumulative mental health problems among violence-affected youth.

### Prevalence of mental health problems

The finding that internalizing problems were more common among girls and externalizing problems more common among boys was expected and is well established in the literature [2,12]. Also, the levels of prevalence rates and the gender ratios are supported by comparable findings in other studies. For example, using standardized clinical interviews, the National Comorbidity Survey Replication-Adolescent Supplement [2] reports similar frequencies and sex ratios to the ones we found in our study with respect to mood (emotional) disorders, ADHD, conduct disorder and substance use disorder for young people of comparable age. However, the high rates of 28.9% for girls and 15.4% for boys of disordered eating in our study needs to be mentioned, as studies examining eating disorders in adolescents usually report far lower rates (from 2.5% to 3.8% in girls and 1.5% to 1.9% in boys [2,68], albeit with similar gender ratios. This discrepancy is probably explained by our measure that captures disordered eating on a subclinical rather than on a clinical level, as indicated above. Nonetheless, it has been shown that those identified with problematic eating constitute a group at risk [67], and the use of this measure has recently been recognized in the literature [46].

### Associations with adolescent violence involvement

In summary, we found that internalizing and externalizing mental health problems, as well as cumulative mental problems, were considerably more common in youth who self-reported violence. This was true for victims, perpetrators, and perpetrating victims. Prevalence rates of mental problems in studies on violence-affected youth are difficult to compare, due to variations in the sampling, the measures applied, and the underlying definitions of violence. Among juvenile offenders in detention, prevalence rates of mental disorders are regularly found to be exceedingly high. For example, different studies report prevalence rates of between 60% and 80% [2,69] for at least one mental disorder. However, such figures depend not least on the number of investigated disorders. Moreover, with respect to violence and adolescent mental health, the value of studies on detained youth is limited because studies rarely distinguish between violent and non-violent offending. In addition, it is likely that imprisonment exacerbates the mental health of detained youth for a variety of other reasons. Also, the non-normative setting does not allow for direct comparisons with an unaffected “control group”. In a study comparable to ours in terms of representativeness and age range, Kaltiala-Heino and colleagues found similar trends in the prevalence of depressive symptoms, anxiety, psychosomatic problems, and substance use (alcohol and other drugs) among Finnish school bullies, victims and bully-victims [68]. However, the prevalence rates in their study were as much as twice as high as in ours. It is difficult to say why. Differences in the measures and/or thresholds applied are conceivable causes, as are cross-national variability.

In multivariate assessment, any kind of violence involvement proved to be a significant risk factor for virtually any mental health problem studied. *Victims* were more likely to internalize problems; i.e. they exhibited higher risks for emotional problems, disordered eating behavior and sleeping problems; this result is consistent with previous findings [5,14,16,23,68]. Unlike in prior studies [28], a history of only victimization was not associated with ADHD in our study. However, Holmberg and Hjern’s study [28] did not distinguish the category of a perpetrating victim. It can thus be assumed that, in their study, at least some of the subjects in the victimization group were indeed simultaneously perpetrators. Nonetheless, in our study victimization was also associated with conduct problems, which might indicate maladaptive coping with experienced aggression [70]. However, we found that victim status was not associated with any kind of substance use; a result that is supported by previous findings on German youth. For example, in the cross-national HBSC study German bullies and school bully-victims scored above average in an alcohol assessment, whereas German victims did not receive such scores [71].

Given the corresponding literature, it was not surprising that the mental health profile of *perpetrators* was characterized by externalizing problems such as conduct problems, ADHD, high alcohol consumption levels, and illegal drug use [9,30,68,71]. However, perpetrators were also found to be more likely than expected to experience internalizing problems, such as anxious and depressive moods, disordered eating behaviors, or sleeping problems, although effect sizes were consistently lower than those of victims or perpetrating victims. Male perpetrators were found to have increased risks for somatic pain, reflecting similar findings by Piko et al. [21]. Therefore, intervention strategies should both target the reduction of externalizing behaviors and address internalizing problems in perpetrators.

The profile of the *perpetrating victim* involved the highest risks for virtually all the mental health problems studied, compared with other violence-affected youth. This was true for emotional problems, disordered eating behaviors and somatic pain (boys), conduct problems, alcohol consumption and illicit drug use. The finding that perpetrating victims are particularly compromised is supported by other findings indicating an exceptional psychosocial vulnerability of this group. For example, in a case control study, dangerously violent adolescents exhibited higher levels of passive violence exposure than matched controls [72]. Likewise, Odgers and colleagues [73] found that a group of violent and delinquent juvenile female offenders with concurrent histories of neglect and exposure to violence exhibited the highest rates of psychiatric disorders compared with an only-delinquent and a low-offending group. Cuevas and collaborators [74] confirmed the high-risk disposition of victimized youth that simultaneously engaged in violent behaviors in a comparison with victimized youth who simultaneously committed property offenses. In line with the results of our study, Logan-Greene and coworkers found that perpetrating victims of youth violence had the most impaired conditions as regards life stress, alcohol and drug use and emotional distress compared with sole perpetrators and sole victims [9].

Translational science posits that traumatic experiences – such as being victimized – may account for individual differences in attributional style, which, in turn, explain differences in aggression [75]. Empirically, individuals who are both anxious and aggressive are found to be likely to attribute hostile intent to the behavior and actions of others (predominantly in ambiguous situations) and are thus more likely both to behave aggressively and to be victimized [76]. Emotional and behavioral dysregulation have been described as causal on the road to peer victimization in aggressive preschool and primary school children [77,78]. In our study, perpetrating victims displayed the highest rates and highest risks for clinical ranges of both emotional and conduct problems. This may indicate that problems with affect regulation, which put younger children at higher risks for simultaneous perpetration and victimization, extend into teen age. However, we were not able to objectify this in our study.

The fact that the likelihood of disordered eating behaviors and somatic pain (boys), sleeping problems, frequent drinking, and illicit drug use is relatively highest among perpetrating victims indicates that the considerable distress of simultaneous victimization and perpetration translates into somatic symptoms and is likely to culminate in the use of psychoactive substances [9]. This pattern applies in particular to female perpetrating victims. These findings are in line with research that highlights the role of trauma in the development of psychopathology and delinquency. Kerig et al. [79] found that posttraumatic stress disorder mediated relations between interpersonal trauma and mental health problems, e.g. depressed/anxious moods, substance use, somatic complaints or suicidal ideation. They also found that the results were stronger among females than among males.

In our study, perpetrating victims exhibited the highest rates of co-occurring mental health problems: 39.1% for girls and 32.1% for boys. Comorbidity rates among detained youth were found to be up to 44.5% for girls and 49.8% for boys [2,80]. The fact that the comorbidity rates among perpetrating victims in a representative sample are almost as high as in a non-normative sample of committed youth should be a particular cause of concern.

Taken together, we found that internalizing and externalizing mental problems were part of the profiles of any kind of violence history in youth – victims, perpetrators and perpetrating victims – which suggests that the occurrence of these categories is not mutually exclusive [69]. Comorbidity may be rooted in a common cause [81]. Being exposed to violence may represent such a cause. For example, the co-occurrence of internalizing and externalizing problems in youth in the context of violence may well be explained within the framework of the cognitive vulnerability-transactional stress depression model [31]. The model posits that broad negative affect initiated by a strong negative interpersonal event – as violence clearly is – accounts for both elevations in depression and externalizing problems. However, a bidirectionality of relations is conceivable and, as our study is cross-sectional, we are unable to confirm causal relationships. On the other hand, it may also be the case that both externalizing and internalizing problems are manifestations of a higher-order general problem factor. Although both types of problem behaviors in part represent unique constructs, Reitz and collaborators showed that higher-order problem structures exist and are stable over time [82]. Nonetheless, the relations to violence exposure have yet to be disentangled.

### Gender patterns

Motivated by observations in earlier non-normative studies [5,44,45,47], one research question of this study was to investigate whether gender cross-over effects would occur in a representative sample. Indeed, we found such reverse associations in the form of five-fold higher risks for conduct problems and up to nine-fold higher risks for illicit drug use in violence-affected girls; there were also increased risks for somatic pain in violent boys, but lower overall prevalence rates for males in our sample. The fact that gender cross-over effects for both boys and girls emerged in a representative sample suggests that this phenomenon is not an artifact of sampling [24]. Nonetheless, the underlying reasons why such effects occur in violence-affected youth are still unknown. Gender research distinguishes between the concepts of gender stereotypes (i.e. people’s beliefs about how the sexes differ or should differ) and gender identity, which can be described as a representation of self in relation to gender categories – including comfort with one’s gender and internalized social pressure to conform to gender stereotypes [83]. Research indicates that pressure to conform to gender norms predicts internalizing problems, more strongly for girls than for boys [84]. It is certainly conceivable that a mismatch between gender stereotypes and gender identity exacerbates maladjusted behaviors in vulnerable girls and boys. This hypothesis is supported by the observation that gender-dysphoric girls are perceived as more aggressive, more disruptive and antisocial by their peers than gender-content girls [85]. On the other hand, highly adversarial interpersonal relationships are one of the gender-specific risk factors for female offending [86]. It is conceivable that it may be functional for some girls to externalize behaviors in the face of a constant threat to their personal safety. Nonetheless, there is a clear need for in-depth research into the specific determinants of reverse gender stereotypes in the context of violence in youth.

### Limitations and strengths

Our study has important limitations. First, because the study is cross-sectional, we are unaware of the causality of the associations. Furthermore, we were unable to present diagnoses according to the DSM or ICD, which are typically obtained from structured diagnostic interviews. However, the use of structured interviews is impractical in the study of large samples. Some of our variables, such as somatic pain, sleeping problems, and frequent drinking, were created following the collection of the sample data. Thus, despite our efforts to ensure optimal operationalization, these measures necessarily remain fuzzy to a certain degree as a result of limited information (for more details, please refer to the methods section). However, we were able to demonstrate a high level of conformity with studies that used clinical interviews in the assessment of adolescent mental health problems – despite imperfect operationalization. The single-item assessment of violent behaviors, as a consequence of restricted resources in large surveys, clearly constitutes a limitation. Also, the violence assessment relied on self-reporting and was not externally validated. However, the self-report method has generally proved to be sufficiently reliable in violence research [87]. Another limitation is that no definition of violence was presented to the participants. Therefore, the participants might have differed in their cognitive concepts. However, research suggests that German students referred to a narrow concept of physical violence in their subjective understanding of the term “violence” [88].

This study adopted the four-fold classification of victims, perpetrators, perpetrating victims and uninvolved youth established by the research on bullying. Although this typology has been successfully confirmed in youth violence research [9,10], it should not be reified. For example, a recent study with this sample challenged the uniqueness of the perpetrating victim category, finding that multiply victimized youth stood out with highest psychosocial risks [89]. In order to warrant sufficiently large cell sizes for the moderator analyses, we could not consider multiply victimized youth as a distinct group in this study because they are small in number and percentage. Yet that does not mean that the present study is compromised, since perpetrating victims were still found to have considerably elevated risks even after multiply victimized youth had been distinguished [89]. An important strength of this study is that we were able to consider a sizable number of mental health problems in a large and nationally representative sample. The results thus allow substantial generalizability to the general population.

## Conclusions

Our study has social-policy and clinical implications, as the results show that violence involvement is strongly associated with the mental health of youth. Both youth violence and poor mental health in young people have been recognized as major public health problems [90,91]. Our results imply that mental-health prevention programs for adolescents must consider violence involvement. Conversely, community-violence prevention efforts should be responsive to the considerable comorbidity of mental health problems in violence-affected youth – particularly in female adolescents. An important clue from our study is that gender stereotypes regarding the mental health of young people can be reversed after experiencing violence and thus need to be carefully reviewed, specifically when new programs are launched. At the clinical level, healthcare professionals should be trained to screen for violence. A recent investigation revealed that primary-care practitioners documented discussions of violence with only 19.4% of seriously violence-affected youth who attended their clinic for a routine or sick visit with their parents [92]. Practitioners should be aware of (and carefully assess for) multiple-risk constellations when either victims, perpetrators or perpetrating victims of violence are referred for treatment. Particular attention should be devoted to female adolescents who report any type of violence involvement, as our study indicates that these adolescents (although fewer in number) may be particularly prone to externalizing behaviors and illicit drug use. This is especially relevant for female perpetrating victims.

## Abbreviations

DSM-IV-TR: Diagnostic and Statistical Manual of Mental Disorders of the American Psychiatric Association (4th ed.) Text revision; ICD-10: International Classification of Diseases 10th Revision; WHO: World Health Organization.

## Competing interests

All authors declare that they have no competing interests.

## Authors’ contributions

RS was responsible for writing the manuscript and performed all the statistical analyses. FP provided specific knowledge and contributed to the analyses and interpretation of the results. Both authors have read and approved the final manuscript.

## Pre-publication history

The pre-publication history for this paper can be accessed here:

http://www.biomedcentral.com/1471-2458/13/628/prepub
